# The covariant structural and functional neuro-correlates of cognitive impairments in patients with end-stage renal diseases

**DOI:** 10.3389/fnins.2024.1374948

**Published:** 2024-04-15

**Authors:** Yuefan Liu, Huiying Wang, Guanchen Sha, Yutong Cao, Yongsheng Chen, Yuanyuan Chen, Jingyi Zhang, Chao Chai, Qiuyun Fan, Shuang Xia

**Affiliations:** ^1^Department of Biomedical Engineering, Medical College, Tianjin University, Tianjin, China; ^2^Tianjin Key Laboratory of Brain Science and Neuroengineering, Tianjin, China; ^3^Haihe Laboratory of Brain-Computer Interaction and Human-Machine Integration, Tianjin, China; ^4^Department of Radiology, School of Medicine, Tianjin First Central Hospital, Nankai University, Tianjin, China; ^5^Intelligent Medical Engineering, Academy of Medical Engineering and Translational Medicine, Tianjin University, Tianjin, China; ^6^Department of Neurology, Wayne State University School of Medicine, Detroit, MI, United States

**Keywords:** multimodal CCA-joint ICA, gray matter volume, fractional anisotropy, amplitude of low-frequency fluctuation, cognitive impairment, end-stage renal disease

## Abstract

**Introduction:**

Cognitive impairment (CI) is a common complication of end-stage renal disease (ESRD) that is associated with structural and functional changes in the brain. However, whether a joint structural and functional alteration pattern exists that is related to CI in ESRD is unclear.

**Methods:**

In this study, instead of looking at brain structure and function separately, we aim to investigate the covariant characteristics of both functional and structural aspects. Specifically, we took the fusion analysis approach, namely, multimodal canonical correlation analysis and joint independent component analysis (mCCA+jICA), to jointly study the discriminative features in gray matter volume (GMV) measured by T1-weighted (T1w) MRI, fractional anisotropy (FA) in white matter measured by diffusion MRI, and the amplitude of low-frequency fluctuation (ALFF) measured by blood oxygenation-level-dependent (BOLD) MRI in 78 ESRD patients versus 64 healthy controls (HCs), followed by a mediation effect analysis to explore the relationship between neuroimaging findings, cognitive impairments and uremic toxins.

**Results:**

Two joint group-discriminative independent components (ICs) were found to show covariant abnormalities across FA, GMV, and ALFF (all *p* < 0.05). The most dominant joint IC revealed associative patterns of alterations of GMV (in the precentral gyrus, occipital lobe, temporal lobe, parahippocampal gyrus, and hippocampus), alterations of ALFF (in the precuneus, superior parietal gyrus, and superior occipital gyrus), and of white matter FA (in the corticospinal tract and inferior frontal occipital fasciculus). Another significant IC revealed associative alterations of GMV (in the dorsolateral prefrontal and orbitofrontal cortex) and FA (in the forceps minor). Moreover, the brain changes identified by FA and GMV in the above-mentioned brain regions were found to mediate the negative correlation between serum phosphate and mini-mental state examination (MMSE) scores (all *p* < 0.05).

**Conclusion:**

The mCCA+jICA method was demonstrated to be capable of revealing covariant abnormalities across neuronal features of different types in ESRD patients as contrasted to HCs, and joint brain changes may play an important role in mediating the relationship between serum toxins and CIs in ESRD. Our results show the mCCA+jICA fusion analysis approach may provide new insights into similar neurobiological studies.

## 1 Introduction

Cognitive impairments (CIs) are commonly seen in end-stage renal disease (ESRD) patients due to neuronal degeneration caused by accumulated un-eliminated uremic toxins in the body (Chai et al., 2019, 2020, 2021; Findlay et al., 2019; Miglinas et al., 2020; Wang et al., 2022, 2023a; Zhang et al., 2023). MRI can provide rich information on brain structure and function and has been demonstrated to be a useful tool to reveal brain changes related to CIs. For example, previous structural MRI (sMRI) and diffusion MRI (dMRI) studies have shown that ESRD patients are characterized by overall atrophy of the gray matter (GM) (Zhang et al., 2013; Wang et al., 2022) and decreased integrity of the white matter (WM) (Zhang et al., 2015). A number of functional MRI (fMRI) studies have reported abnormal activities in the default mode network (DMN) (Ni et al., 2014; Ma et al., 2015; Cao et al., 2022). It is known that normal cognition relies on the collaborative wellness of both structural and functional aspects of the brain, and any deviation from this normality may be characterized by a disease-specific pattern of structural and functional abnormalities that are intrinsically related. However, previous MRI studies in ESRD usually take an approach to analyze different types of MRI data (i.e., T1w, DTI, and fMRI) separately, providing an isolated view of the structural or functional characteristics, lacking an effective strategy to investigate the underlying association between them.

Recently, data-driven multimodal analysis methods have been proposed to analyze multimodal data concurrently to uncover the joint alterations that exist between various aspects of brain characteristics (Lottman et al., 2018; Grecucci et al., 2023; Khalilullah et al., 2023). In other words, the multimodal joint analysis methods can reveal associative group differences between different modalities, or covariant abnormalities, allowing for a joint analysis of imaging data of different nature or dimensions, which is hard to achieve otherwise. Among all these methods, the combination of multimodal canonical correlation analysis (mCCA) and joint independent component analysis (jICA), namely, mCCA+jICA (Sui et al., 2011), has been proved to be useful in gaining a deeper understanding of various neurological diseases, such as schizophrenia, mild cognitive impairment, bipolar disorder, catatonia, neural correlates of cognitive control, and obsessive-compulsive disorder, etc. (Sui et al., 2013a; He et al., 2017; Lerman-Sinkoff et al., 2017; Hirjak et al., 2020; Liang et al., 2021). Specifically, the mCCA+jICA method is composed of two major steps. The mCCA step can establish connections between data of different modalities by maximizing the correlation between the resulting canonical variants from each modality (Correa et al., 2008). The jICA step then linearly decomposes the resulting multi-modal canonical variants jointly to achieve the maximally independent sources, i.e., joint ICs.

Along with the efforts to understand how toxins may induce cognitive impairments, previous studies have indicated that the accumulation of uremic toxins can interfere with the central nervous system in ESRD patients, leading to neurotoxicity (Hamed, 2019), and has a significant impact on cognitive function (Liabeuf et al., 2021). Fusion analysis methods can provide insights into joint brain changes using imaging data, but the relationship between these cross-modality covariant abnormalities and cognitive impairments induced by the accumulated un-eliminated uremic toxins in ESRD patients has been unexplored so far. There is evidence that a relationship between covariant abnormalities and cognitive decline exists in diseases such as subjective cognitive decline and schizophrenia (Sui et al., 2013a; Liang et al., 2021). Therefore, we hypothesized that the covariant abnormalities may play a role in mediating the relationship between CI and toxins. A mediation effect analysis was performed to investigate the role of brain abnormalities across modalities in the pathways that toxins contribute to CI, providing a more comprehensive explanation for the results of the fusion analysis at the level of the pathophysiological mechanisms of cognitive impairment.

In this study, we first employed the mCCA+jICA algorithm to jointly analyze sMRI, DTI, and resting-state functional MRI (rs-fMRI) data, to explore the potential covariant abnormalities in brain structure and function associated with CI in ESRD. Subsequently, we performed a mediation effect analysis to investigate the mediating role of joint brain changes in the relationship between CI and uremic toxins to gain a deeper understanding of the pathogenic mechanisms of CI in ESRD. To the best of our knowledge, this is the first attempt to focus on the covariant structural and functional changes and their associations with CI and potentially with uremic toxins in ESRD patients.

## 2 Materials and methods

### 2.1 Participants

The study was approved by the ethics committee of Tianjin First Central Hospital, and all participants signed informed consent before MRI examinations. One hundred ESRD patients were recruited from the Department of Hemodialysis of the hospital. All ESRD patients had hemodialysis for more than 3 months, and dialysis was performed three times a week for 4 h each time. Eighty-two healthy controls (HCs) were recruited from the hospital or local community. Inclusion criteria for all subjects were: (1) aged over 18 years and righted hand; (2) no drug abuse, neurological diseases (e.g., epilepsy, head trauma/contusion, cerebral hemorrhage), or other diseases or treatments that may affect the central nervous system (e.g., liver or kidney transplantations); (3) able to complete the neurocognitive evaluation; and (4) no congenital structural abnormalities in the brain. Among all subjects, 34 were excluded due to inconsistent imaging parameters, and another 6 were excluded due to poor imaging data quality, yielding a total of 78 ESRD patients (41 males and 37 females, age range from 18 to 75 years old, mean 45.7 ± 13.8 years old) and 64 HCs (27 males and 37 females, age range from 22 to 64 years old, mean 42.5 ± 11.5 years old) remaining in the study. A mini-mental state examination (MMSE) was performed on all enrolled participants to evaluate their cognitive status (Table 1).

**Table 1:** Demographic and clinical information of participants.

	ESRD (*n* = 78)	HCs (*n* = 64)	*p*-value
Age (years old)	45.7 ± 13.8	42.5 ± 11.5	0.15^*^
Gender (M/F)	41/37	27/37	0.22^#^
Education level (years)	13.1 ± 2.9	13.8 ± 1.9	0.11^*^
Urea (mmol/L)	25.8 ± 8.7	–	–
Creatinine (mmol/L)	888.3 ± 305.7	–	–
Uric acid (umol/L)	352.3 ± 91.2	–	–
Sodium (mmol/L)	139.3 ± 3.4	–	–
Potassium (mmol/L)	5.1 ± 0.7	–	–
Phosphate (mmol/L)	2.0 ± 0.6	–	–
Alkaline phosphatase (U/L)	87.1 ± 77.6	–	–
Parathyroid hormone (U/L)	281.1 ± 359.9	–	–
*β*_2_-microglobulin (mg/L)	33.1 ± 5.9	–	–
MMSE score	26.9 ± 2.9	29.5 ± 0.9	<0.001^*^

HCs, healthy controls; ESRD, end-stage renal disease; MMSE, mini-mental state examination.

*Two independent sample *t* test; #chi-square test.

### 2.2 Data acquisition

All MRI data were acquired on a 3 T MRI Siemens Tim Trio system with an 8-channel head coil. The imaging parameters are as follows. fMRI: resting-state fMRI images were collected using an echo-planar imaging (EPI) sequence, slices were carefully oriented along the anterior commissure–posterior commissure line, repetition time (TR) = 3 s, echo time (TE) = 30 ms, field of view (FOV) = 175 × 238 mm^2^, flip angle = 90°, slice thickness = 3 mm, number of slices = 38, matrix size = 50 × 64, acquisition time = 11 min 30 s, voxel size = 3.4 × 3.4 × 3.4 mm^3^. sMRI: T1-weighted data were acquired using a magnetization-prepared rapid gradient-echo (MP-RAGE) sequence with the following parameters: TR/TE/inversion time (TI) = 1,900/2.52/900 ms, FOV = 258 × 256 mm^2^, flip angle = 9°, slice thickness = 1 mm, number of slices = 176, matrix size = 256 × 256, acquisition time = 211 s, and voxel size = 1.0 × 1.0 × 1.0 mm^3^, bandwidth = 170 Hz/pixel. DTI: diffusion data were acquired using an axial single-shot spin-echo EPI sequence, TR = 10.5 s, TE = 103 ms, FOV = 239 × 240 mm^2^, slice thickness = 1.8 mm, number of slices = 38, matrix size = 128 × 128, acquisition time = 215 s, voxel size = 1.8 × 1.8 × 1.8 mm^3^, b = 0 s/mm^2^, 1,000 s/mm^2^ (30 directions), 2,000 s/mm^2^ (30 directions).

### 2.3 Data analysis

**Figure 1:**
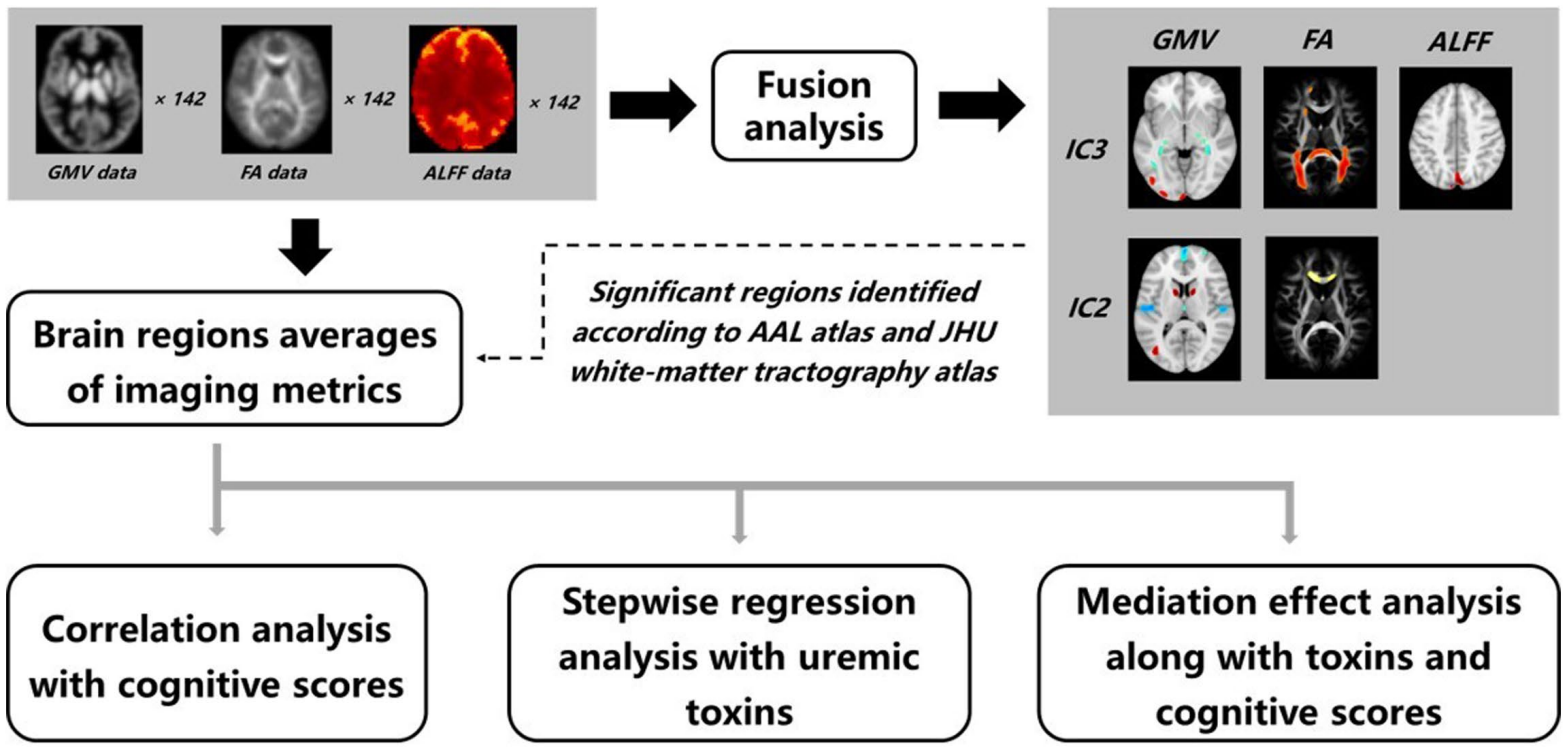
Workflow of data processing and analysis. A fusion analysis on GMV, FA, and ALFF data was performed to obtain joint group-discriminative independent components between the ESRD and HCs groups. The abnormal brain regions were identified accordingly, and averages of GMV, FA, and ALFF were calculated. The obtained imaging metrics were then fed into a series of statistical examinations, including correlation, stepwise regression, and mediation effect analysis.

The workflow of data processing and analysis is shown in Figure 1, which includes the fusion analysis of multimodal data, obtaining regional values of GMV, FA, and ALFF, and statistics analysis, etc., as described below.

#### 2.3.1 Preprocessing and feature extraction

The preprocessing of sMRI images was performed by Advanced Normalization Tools (ANTs) (Avants et al., 2011) and FreeSurfer software (Fischl, 2012). The N4 bias field correction was performed (Tustison et al., 2010) on original T1w data to filter out low-frequency fluctuation artifacts. Then, the corrected T1w images were nonlinearly registered to the Montreal Neurological Institute (MNI) template using the “antRegistrationSyNQuick.sh” command in ANTs with the deformation field information preserved. To obtain the gray matter images, different brain tissues (cortex, subcortex, white matter, and cerebrospinal fluid) in T1w images were segmented (Dale et al., 1999) and labeled by the “recon-all” command in FreeSurfer and then based on the labeling information, the volume fractions maps of GM in the native space were calculated by the “mri_compute_volume_fractions” command, i.e., the numerical value of each voxel indicated the proportion of gray matter. The GM volume fractions maps were transformed into the 1 × 1 × 1 mm^3^ MNI standard space by applying the deformation field information and then multiplied by the Jacobian determinant images calculated from the deformation field information to preserve the gray matter volume (GMV) of native space (Good et al., 2001). Finally, the GMV images were smoothed by an 8 mm full-width at half-maximum (FWHM) Gaussian kernel. The workflow of sMRI data processing is illustrated in Supplementary Figure S1.

The dMRI data images were preprocessed using the FMRIB Software Library (FSL) (Jenkinson et al., 2012). For the diffusion-weighted images, the “eddy_correct” command was used to correct the image distortion caused by the eddy current, and the brain mask was created by extracting the brain tissues of the volume without diffusion-weighted (b0 images) to provide the calculation range of tensor reconstruction. Then, the FA maps were calculated by diffusion tensor reconstruction with the “dtifit” command. Before the registration of FA maps, b0 images were transformed into native T1w space, followed by the transformation of T1w images into standard space. Subsequently, the FA maps were registered to the 2 × 2 × 2 mm^3^ MNI standard space utilizing both the affine transformation matrix and deformation field information generated during the above-mentioned two-step transformations. Finally, the FA images were smoothed with an 8 mm FWHM Gaussian kernel. The workflow of dMRI data processing is illustrated in Supplementary Figure S2.

The preprocessing of rs-fMRI images aimed to obtain ALFF maps using the Analysis of Functional NeuroImages (AFNI) software. Specifically, the first five time points of the original fMRI data were removed to reduce the influence of unstable signal acquisition at the beginning. The remaining 225 time points were included for slice-timing correction and motion correction. Then, the signals from the 24 motion-related parameters (six motion correction parameters, derivative, and their quadratic terms) and the averages of WM and cerebrospinal fluid in time series were considered as nuisance signals and removed by multiple linear regression. A bandpass filter ranging from 0.008 to 0.08 Hz was applied to reduce the extremely low-frequency drift and high-frequency physiological noise, aligning with the frequency of the blood oxygen level-dependent signal. The global signal regression was performed to remove non-neuronal sources of global variance such as respiration and movement (Yan et al., 2013). Before calculating the ALFF maps, data were spatially smoothed with an 8 mm FWHM Gaussian kernel. Subsequently, the power spectrum of the time series for each voxel was acquired through the Fast Fourier Transform (FFT), and the amplitude was obtained by taking the square root of the spectral value of each discrete frequency point in the power spectrum, and then the discrete amplitude sequence with frequencies between 0.008–0.08 Hz was averaged to obtain the ALFF value for each voxel. Finally, the ALFF maps were registered to the 3 × 3 × 3 mm^3^ MNI standard space. The workflow of rs-fMRI data processing is illustrated in Supplementary Figure S3.

#### 2.3.2 Multimodal CCA- joint ICA

**Figure 2:**
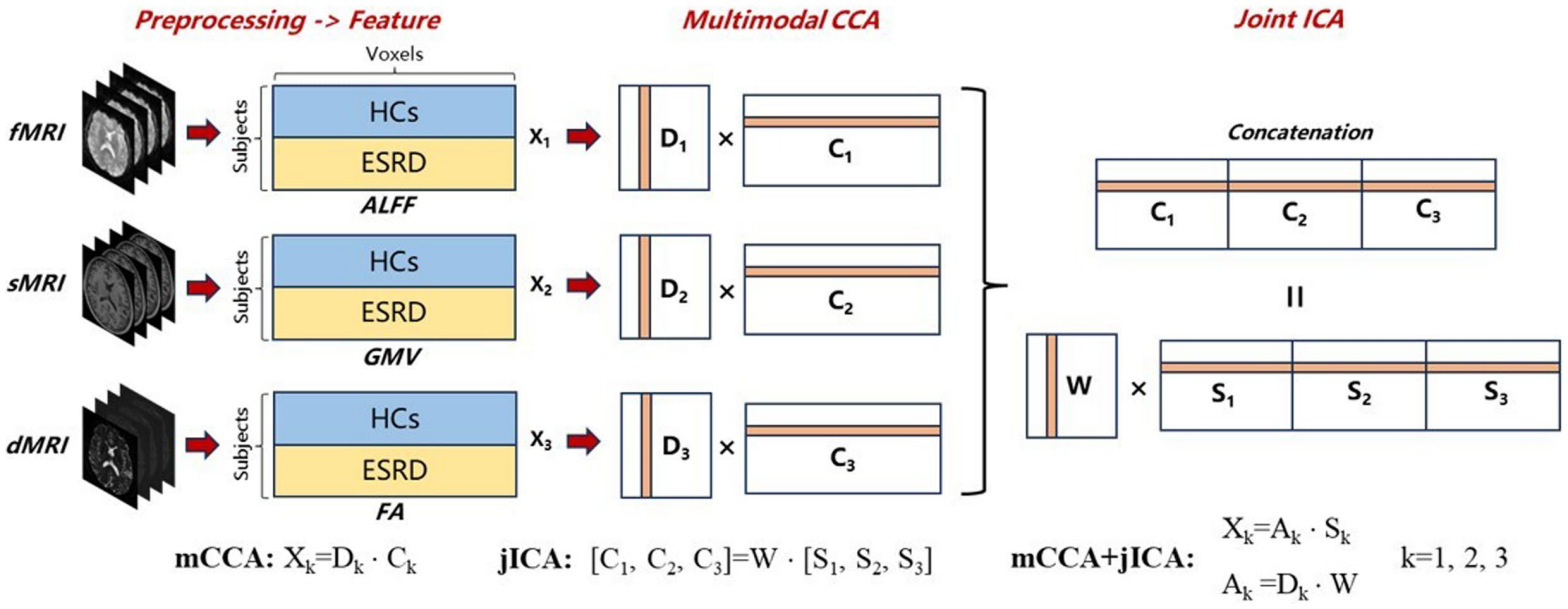
Flowchart of mCCA + jICA for fusion analysis of fMRI, sMRI, and dMRI images. First, preprocessing and feature extraction were conducted on fMRI, sMRI, and dMRI images to obtain ALFF, GMV, and FA feature images, which were then reshaped into feature matrices X_k_ (k = 1,2,3). Following normalization and dimensionality reduction, mCCA decomposed the reduced X_k_ into canonical variant matrices D_k_ and their corresponding source matrices C_k_. Subsequently, jICA was applied to the concatenated source matrix [C_1_, C_2_, C_3_] to decompose a shared mixing matrix W and joint-independence component matrices S_k_. The final mCCA+jICA results can be represented as X_k_ = A_k_ × S_k_, where the mixing matrices A_k_ were obtained by the product of D_k_ and W.

The Fusion ICA Toolbox (FIT^1^) in MATLAB was used to perform the fusion analysis on GMV, FA, and ALFF images for 142 subjects (Sui et al., 2013a). The goal of mCCA+jICA is to identify an equivalent number of independent sources for each modality. These sources referred to as ICs, can be linearly combined to reconstruct images for each subject in the dataset. This process can be represented as X_k_ = A_k_ × S_k_, where X represents the dataset, A is the mixing matrix, S is the source matrix, and k is the index of the dataset. The flowchart of mCCA+jICA is shown in Figure 2.

First, the three-dimensional imaging data within brain masks for each subject was reshaped into one-dimensional row vectors. These vectors were then stacked to form the two-dimensional feature matrices. Each of the three modalities corresponds to its own feature matrix X_k_ (k = 1,2,3, dimension = [number of subjects] × [number of voxels]). Then, all the data matrices were normalized to have equal average sum-of-squares (computed across all participants and all voxels). Normalization was necessary because ALFF, GMV, and FA data have largely different ranges (Sui et al., 2013a). After normalization, a minimum description length (MDL) criterion (Li et al., 2007) was used to estimate the number of ICs for each dataset, determined to be 12 in this experiment. Principal component analysis (PCA) was conducted on the normalized matrices to achieve dimensionality reduction, aiming to mitigate the influence of noise and cross-correlation of voxel signals on fusion analysis (Sui et al., 2013a). The dimensionality of the reduced matrices Y_k_ (k = 1,2,3) was [number of subjects] × 12, preserving 93, 98, and 99% of the variance in the GMV, FA, and ALFF datasets, respectively.

The mCCA algorithm first decomposed Y_k_ into canonical variant matrices D_k_ and their corresponding source matrices C_k_. These source matrices were associated with each other through the correlation of canonical variants located in the same column of D_k_. Subsequently, jICA was applied to the concatenated source matrix [C_1_, C_2_, C_3_] to decompose a shared mixing matrix W and joint-independence component matrices S_k_. The quality of the jICA decomposition results was automatically assessed by the ICASSO software in FIT, and the most stable results were selected as final outputs to ensure reliability (Himberg et al., 2004). Finally, the mixing matrices A_k_ were obtained by the product of D_k_ and W, indicating the proportion of ICs in the reconstruction of the subject’s images. Therefore, the ICs that can reveal group differences can be identified by testing the differences in mixing coefficients between the HC and ESRD groups. Vectors within the same row across matrices S_k_ correspond to the same IC, each comprising spatial maps of GMV, FA, and ALFF. In ICs with group differences, significant brain regions identified in spatial maps may indicate potential structural or functional abnormalities in ESRD. Additionally, the three spatial maps within the same IC are associated through the correlation among their mixing coefficients (Sui et al., 2011), suggesting the potential covariant abnormalities of structure and function in ESRD.”

#### 2.3.3 Identification of significant ICs

Analysis of covariance (ANCOVA) was performed on the mixing coefficients with adjustment for age, gender, and education levels to identify the ICs that showed differences between HCs and ESRD, followed by multiple comparisons using false discovery rate (FDR) correction (Benjamini-Hochberg Method, *q* = 0.05, *p* < 0.0138). The ICs that can distinguish the two groups with statistical significance in two or more modalities simultaneously are referred to as joint group-discriminative ICs (Sui et al., 2013b). The significant joint ICs were transformed into maps of z-scores and a threshold at |Z| ≥ 2 was set to only show the brain regions with greater GMV/FA/ALFF values in ICs. The Automated Anatomical Labeling (AAL) brain atlas (Tzourio-Mazoyer et al., 2002) was used to report the significant brain regions in GMV and ALFF of ICs. Fiber tracking seeded from clusters of significant ICs on FA maps and terminated by the significant clusters of GMV and ALFF in the same IC was performed (see Supplementary material for the details of fiber tracking). This approach allows for the identification of abnormal fiber tracts with structural connections to brain regions exhibiting abnormal GMV or function. Not only does this method facilitate a more precise assessment of fiber tract information within clusters of FA_IC, but it also provides a potential explanation for covariation abnormalities between white matter structure and gray matter volume or function. The Johns Hopkins white matter (WM) tractography atlas (Hua et al., 2008) in FSL was used to report the fiber tracking results.

#### 2.3.4 Mediation effect analysis and its pre-analysis: imaging metrics, uremic toxins, and cognitive scores

Before investigating the mediating relationships among imaging metrics, uremic toxins, and cognitive scores, it is essential to identify potential nodes in the mediating pathways. These nodes refer to brain regions whose imaging metrics are significantly associated with cognitive function, as well as the types of toxins contributing to brain changes. This identification was accomplished through partial correlation analysis for brain regions and stepwise regression analysis for toxin types. All analyses were performed on data from the ESRD group.

First, the averages of GMV, FA, and ALFF in ESRD groups were calculated for brain regions identified from the maps of significant ICs, among which the averages, referred to as imaging metrics, were calculated from the complete brain regions in the atlases. Then, a partial correlation analysis was performed to examine the relationship between the imaging metrics and the MMSE scores with age, gender, and education levels as covariates. Furthermore, to reduce the influence of hypertension factors on the results, whether patients with ESRD had hypertension (hypertension status) was included as the fourth covariate in the partial correlation analysis. The correlation results were subjected to FDR correction using the Benjamini-Hochberg method (*q* = 0.05, *p* < 0.020). Subsequently, a stepwise regression analysis was performed to investigate the relationship between uremic toxins and brain changes indicated by the imaging metrics of brain regions identified in FA, GMV, and ALFF. Some uremic toxins that are thought of as indicators of disease progression in clinical practice (Viggiano et al., 2020; Liabeuf et al., 2021; Rosner et al., 2021) were included in the analysis, such as urea, creatinine, uric acid, sodium, potassium, phosphate, alkaline phosphatase, parathyroid hormone, and β_2_-microglobulin.

Based on the results of partial correlation analysis and stepwise regression analysis, the mediation effect analysis was performed to investigate the relationship among the brain changes, uremic toxins, and CI using the PROCESS macro tool v3.5 in SPSS. Bias-corrected 5,000 bootstrapping samples were performed to measure the 95% confidence interval to estimate direct, indirect, and total effects. Age, gender, education levels, and hypertension status were used as covariates regressed on the mediators and outcome simultaneously.

## 3 Results

### 3.1 Demographics and clinical information in ESRD and HCs

No significant differences in age, gender, or education levels between ESRD and HCs were found (all *p* > 0.05). The MMSE scores between ESRD and HCs showed significant differences (*p* < 0.001) (Table 1).

### 3.2 Group-discriminative independent components

**Figure 3:**
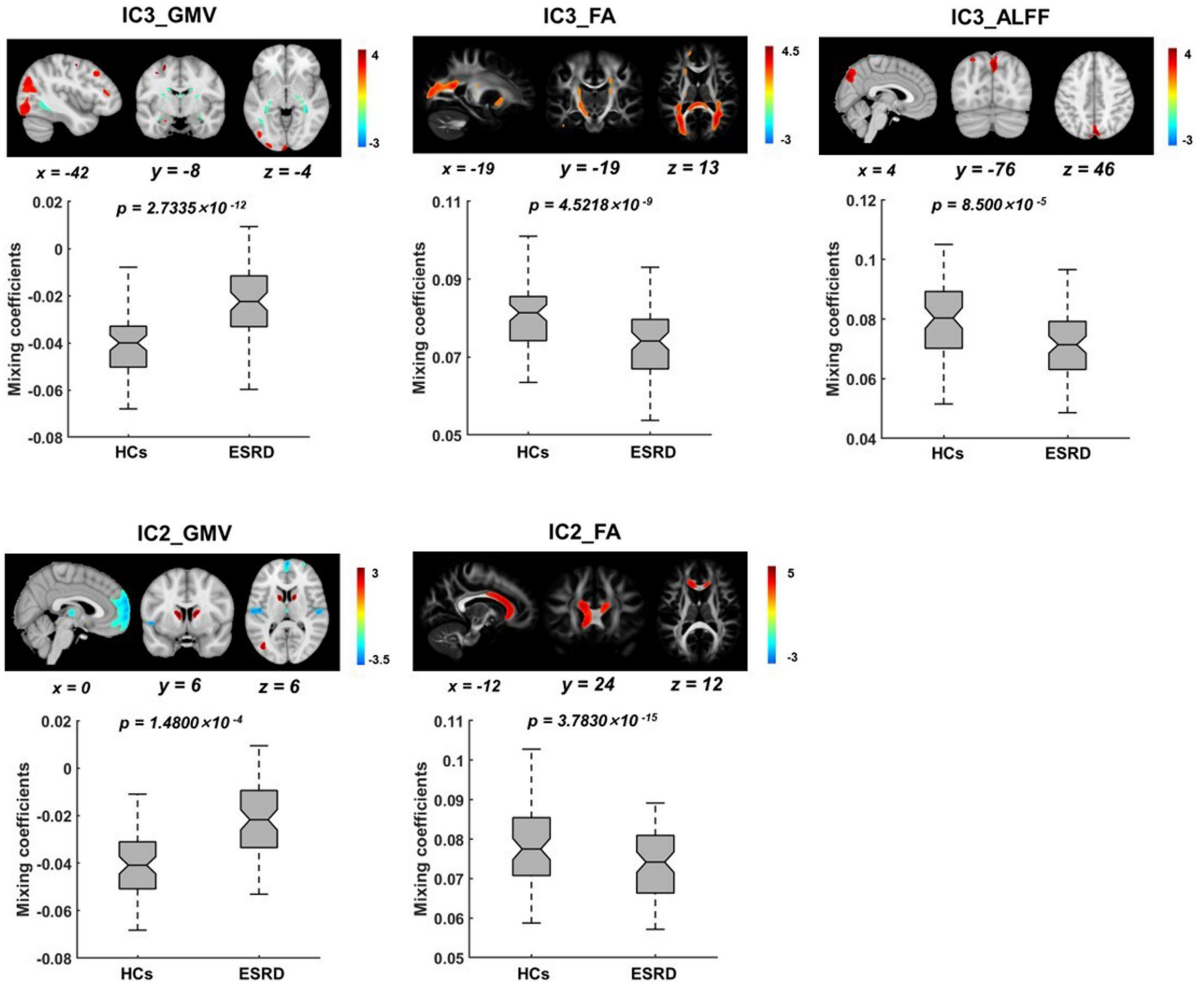
Spatial distributions of the significant joint group-discriminative ICs and their corresponding mixing coefficients. IC3 exhibits covariant abnormalities across all three modalities of GMV **(A)**, FA **(B)**, and ALFF **(C)**, while IC2 exhibits covariant abnormalities across only GMV **(D)** and FA **(E)**. The IC maps were presented in Z scores, with a threshold of |z| ≥ 2, and all *p*-values were FDR-corrected.

Five ICs with significant differences in brain imaging indicators between ESRD and HCs were found by the two-sample *t*-tests of the mixing coefficients. However, among these significant ICs, only two were joint group-discriminative ICs, while the remaining ICs exhibited group differences only in scattered single modalities, failing to provide cross-modal covariant abnormalities information. As a result, two representative joint group-discriminative ICs (i.e., IC3 and IC2) were chosen for further investigation. Specifically, IC2 was able to distinguish between HCs and ESRD in both GMV and FA (Figure 2), while IC3 was found to be group-discriminative in all three modalities (Figure 3). The remaining significant unimodal ICs were GMV_IC7, GMV_IC10 and ALFF_IC11 (see Supplementary material for details of other group-discriminative ICs).

**Figure 4:**
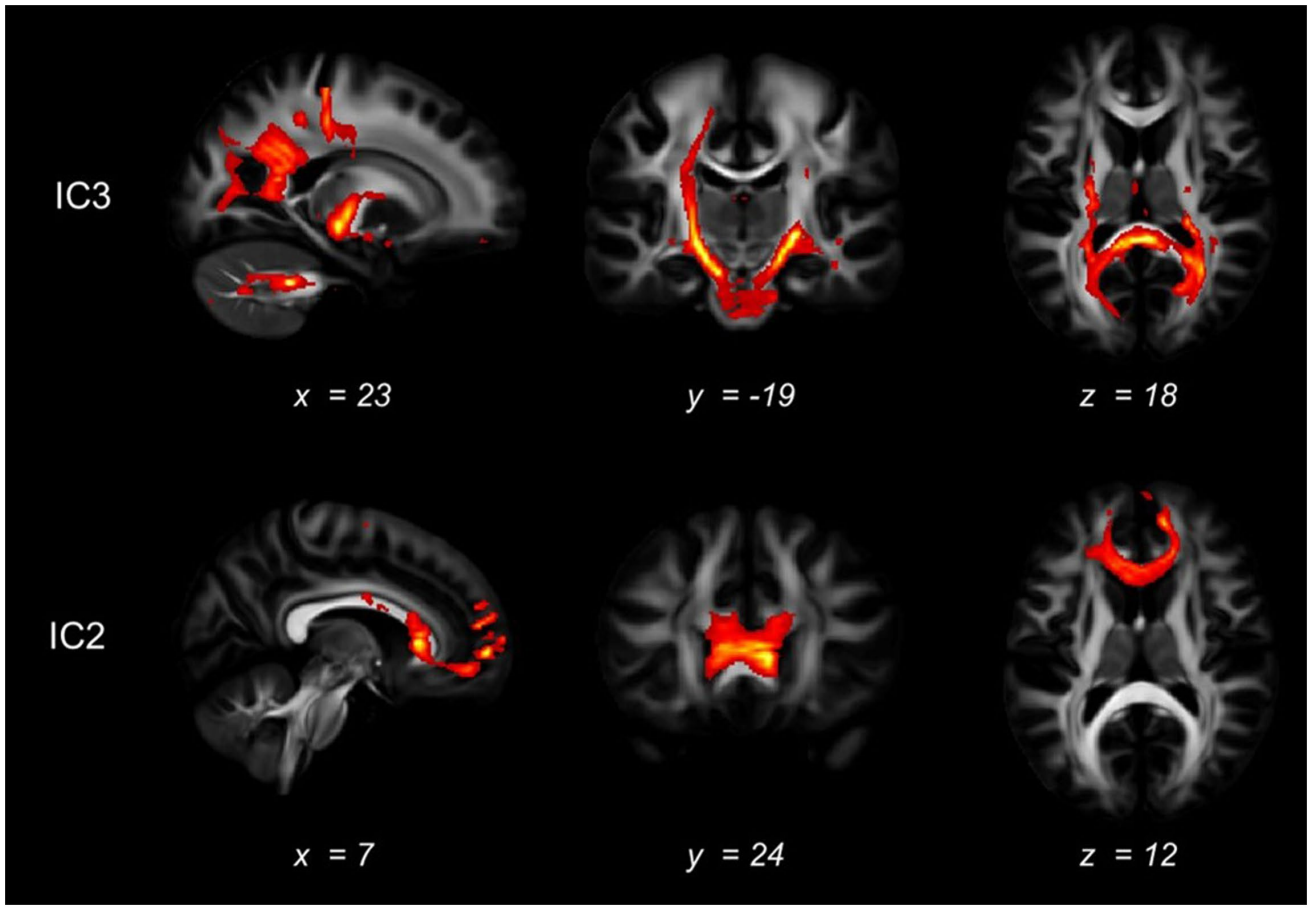
White matter tracts connected with the significant brain regions. The white matter that may be connected with the regions identified as abnormal in the mCCA+jICA method was shown, as indicated by diffusion tractography. The first row displayed the fiber tracking results of the abnormal white matter areas in the IC3, while the second row showed the fiber tracking results in the IC2.

In IC3, as shown in Figure 3, significant brain regions in ALFF involve the precuneus, superior parietal gyrus, cuneus, and superior occipital gyrus. Significant GMV regions were found in the precentral gyrus, superior occipital gyrus, middle occipital gyrus, superior temporal gyrus, middle temporal gyrus, inferior temporal gyrus, middle frontal gyrus, lingual gyrus, hippocampus, parahippocampal gyrus, and medial frontal gyrus. Pertinent fiber tracts as identified by associated significant FA ICs in the fiber tracking analysis included corticospinal tract (CST), inferior frontal occipital fasciculus (IFOF), and forceps major (Figure 4).

In IC2, significant GMV regions include the dorsolateral prefrontal cortex, caudate nucleus, medial superior frontal gyrus, orbitofrontal cortex, middle occipital gyrus, and thalamus (Figure 3). Associated fiber tracts may include the forceps minor, which showed structural connections with the medial superior frontal gyrus, bilateral dorsolateral prefrontal cortex and the orbitofrontal cortex (Figure 4).

### 3.3 Correlations between imaging metrics and cognitive scores

**Figure 5:**
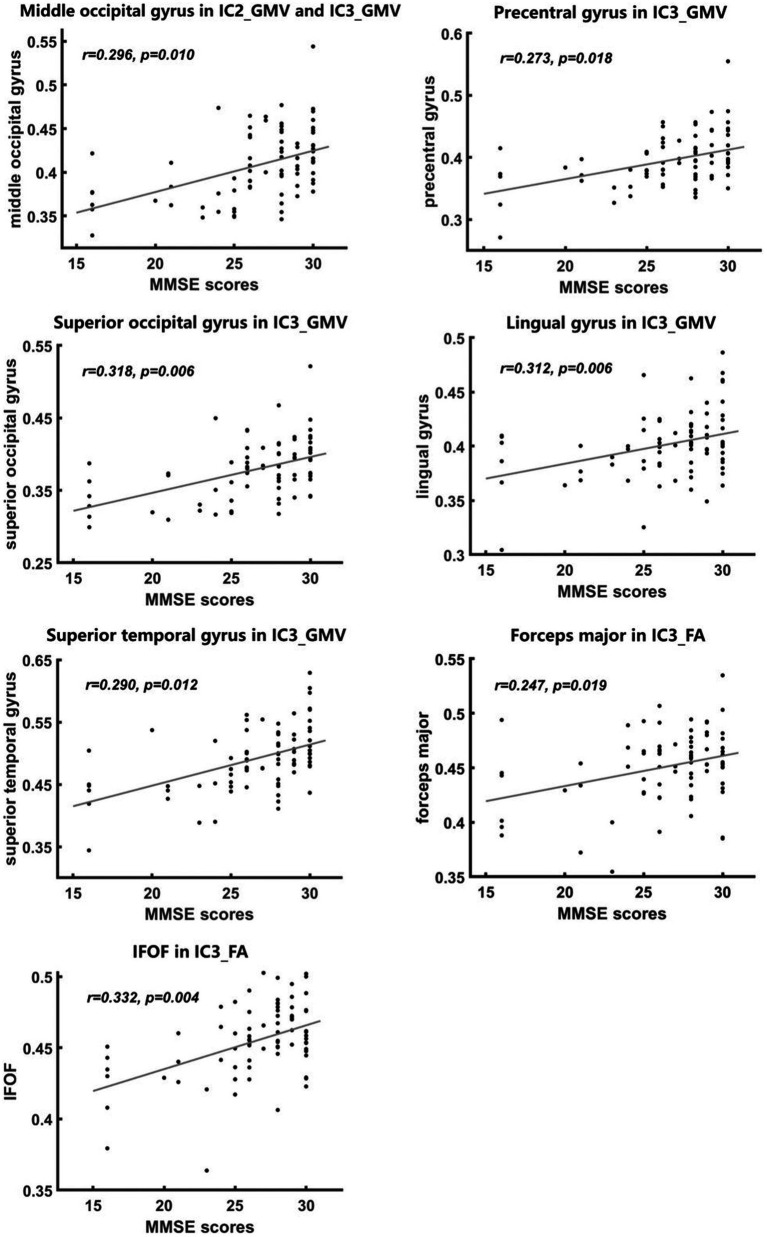
Correlations between imaging metrics and MMSE scores. Significant positive correlations between MMSE scores and FA and GMV values were found in some abnormal brain regions of the IC3 and IC2 findings **(A–G)**, with age and gender controlled as covariates. All *p*-values were FDR-corrected for multiple comparisons.

We found significant positive correlations between the volumes of middle occipital gyrus, superior temporal gyrus, superior occipital gyrus, precentral gyrus in IC3 and MMSE scores and between the volumes of middle occipital gyrus in IC2 and MMSE scores (Figure 5).

We also found significant positive correlations between FA values of IFOF and forceps major in IC3 and MMSE scores (Figure 5).

### 3.4 Uremic toxins associated with brain changes in ESRD

In IC3, phosphate and β2-microglobulin were the independent contributing factors to the FA value of forceps major (βPhosphate = −0.264, *p* = 0.013; ββ2-microglobulin = −0.334; *p* = 0.002). β2-microglobulin was the independent contributing factor to the FA value of CST (ββ2-microglobulin = −0.304; *p* = 0.007). β2-microglobulin and urea were the contributing factors to the FA value of IFOF (ββ2-microglobulin = −0.271, *p* = 0.014; βurea = −0.234, *p* = 0.033). Sodium was the contributing factor to the ALFF value of precuneus (βsodium = 0.244; *p* = 0.031), cuneus (βsodium = 0.226; *p* = 0.047), superior occipital gyrus (βsodium = 0.226; *p* = 0.047) (Table 2).

**Table 2:** Factors contributing to brain changes (i.e., FA, GMV, and ALFF) revealed by the stepwise regression analysis.

Imaging metrics	Clinical risk factors	Standardized coefficients	95% confidence interval	Partial correlation	VIF	*p-*value
FA of forceps major in IC3	Serum phosphate	−0.264	(−0.026, −0.003)	−0.282	1.029	0.013
	*β*_2_-microglobulin	−0.334	(−0.003, −0.001)	−0.348	1.029	0.002
FA of CST in IC3	*β*_2_-microglobulin	−0.304	(−0.002, −0.000)	−0.304	1.000	0.007
FA of IFOF in IC3	*β*_2_-microglobulin	−0.271	(−0.002, −0.000)	−0.279	1.002	0.014
	Urea	−0.234	(−0.001, −0.000)	−0.244	1.002	0.033
ALFF of precuneus	Sodium	0.244	(0.621, 12.777)	0.244	1.000	0.031
ALFF of cuneus	Sodium	0.226	(0.122, 17.319)	0.226	1.000	0.047
ALFF of superior occipital gyrus	Sodium	0.226	(0.122, 17.319)	0.226	1.000	0.047

VIF, variance inflation factor; FA, fractional anisotropy; GMV, gray matter volume; ALFF, amplitude of low-frequency fluctuation; CST, corticospinal tract; IFOF, inferior frontal occipital fasciculus.

In IC2, no predictive factors were found for FA or GMV in any abnormal brain regions.

### 3.5 Mediation analysis among uremic toxins, imaging metrics, and cognitive scores

**Figure 6:**
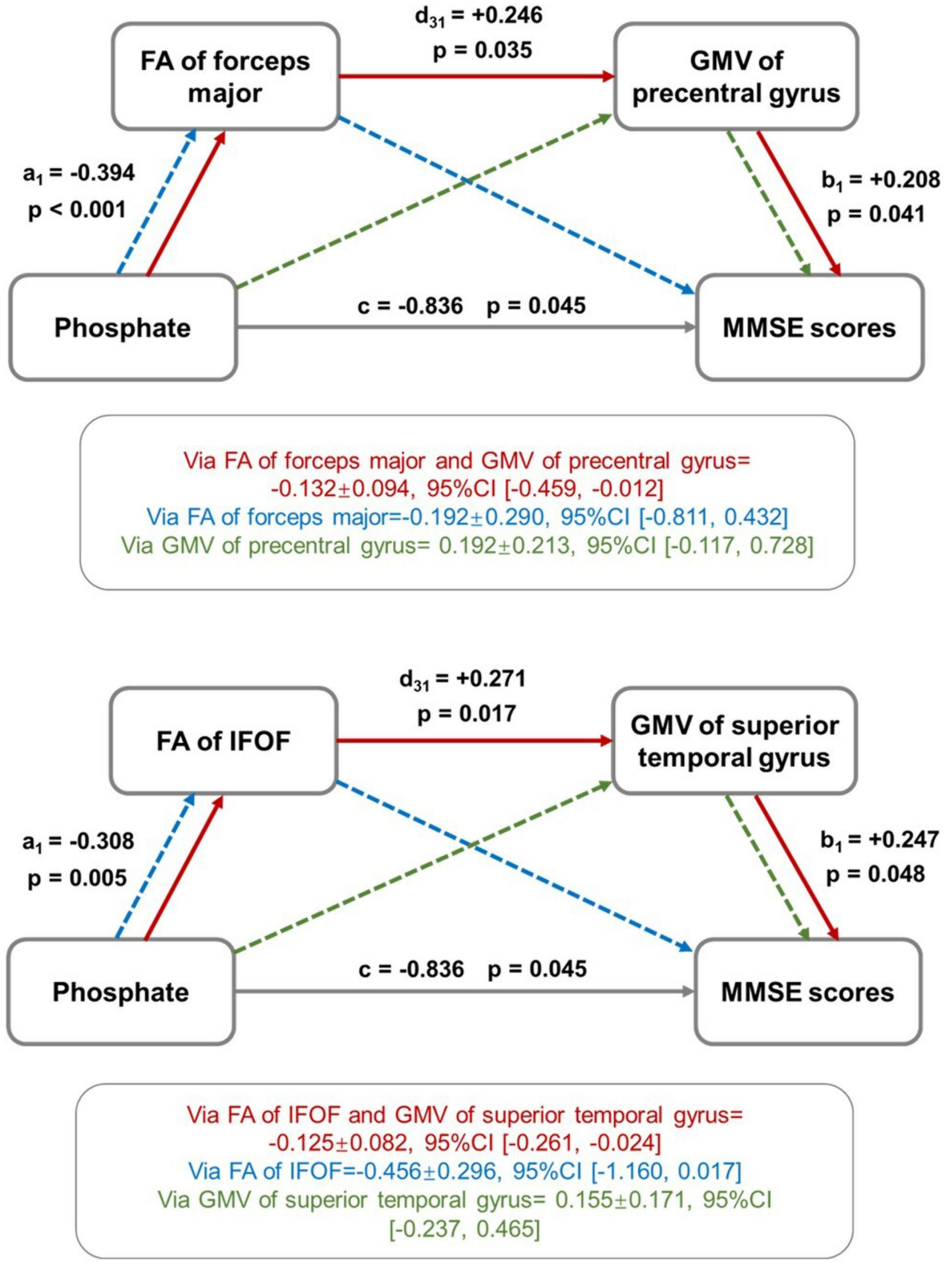
Mediation effect analysis among imaging metrics, uremic toxins, and cognitive scores. The FA and GMV values of selected brain regions were found to play a role in mediating the relationship between serum phosphate and MMSE scores. The red solid line showed the mediation effect direction: “serum phosphate → FA value → GMV value → MMSE scores,” indicating that the increased level of serum phosphate induced the decreased FA values, which further induced the decreased GMV values, yielding the ultimate results of low MMSE scores. The green and blue dot lines represent trends of mediation effects that are not statistically significant.

In IC3, the FA value of forceps major and GMV of the precentral gyrus, FA value of IFOF and GMV of superior temporal gyrus mediated the relationship between serum phosphate and MMSE scores (Figure 6).

In IC2, no significant mediation analysis results were found among the uremic toxins, brain changes, and CI.

## 4 Discussion

This study aimed to explore brain covariant abnormalities associated with ESRD through the fusion analysis of multimodal MRI and investigate the relationship between neuroimaging findings and cognitive impairments and uremic toxins. Two joint group-discriminative ICs that can reflect cross-modality covariant abnormalities were found, i.e., IC3 and IC2, where IC3 exhibited significant group differences among three modalities (ALFF, GMV, and FA), and IC2 showed group-discriminative patterns in GMV and FA. Our study revealed a serial mediation runs from “phosphate → FA changes → GMV changes → cognitive impairment,” indicating that the covariant abnormalities played a mediating role in the pathways of serum phosphate correlated to the severity of CI in ESRD patients.

### 4.1 Covariation of brain structure and function

In IC3, three modalities showed significant changes in ESRD compared to HCs. Among them, the joint alterations of the temporal lobe, parahippocampal gyrus, and hippocampus in GMV and precuneus in ALFF indicated the abnormalities of DMN in ESRD (Raichle and Snyder, 2007; Esposito et al., 2009; Whitfield-Gabrieli and Ford, 2012; Raichle, 2015). The medial temporal lobe is involved in both emotion and memory processes (Gluth et al., 2015), and the memory network formed by the medial temporal lobe, hippocampus, and parahippocampal gyrus in DMN plays a vital role in the coordination of episodic memory and scene-related cognitive functions (Valenstein et al., 1987; Song et al., 2011). Several studies have shown atrophy in these brain regions in ESRD, leading to alterations in both the structure and function of the DMN (Qiu et al., 2014; Zheng et al., 2022; Jiang et al., 2023). The parahippocampal gyrus is considered to establish the connection between the medial temporal lobe memory system and the DMN (Ward et al., 2014), involved in transmitting information such as spatial details and memory encoding to the hippocampus. The hippocampus is responsible for integrating information and the formation and storage of memories. Studies have shown that renal disease can induce neuronal death in the hippocampus (Kim et al., 2014), resulting in the loss of memory that was originally stored in the organized synaptic connections between neurons (Viggiano et al., 2020). Therefore, the observed covariant abnormalities in these brain regions may be associated with the decline in memory abilities in ESRD. Furthermore, as the core node of the DMN, the precuneus is involved in self-referential processing, episodic memory, and executive functions (Fletcher et al., 1995). The long-term accumulation of neurotoxins significantly impacts the structure and spontaneous brain activity of the precuneus in ESRD patients, leading to a decline in psychomotor speed and memory abilities.

The superior parietal gyrus and superior occipital gyrus in ALFF, temporal lobe in GMV, and IFOF in FA are a group of joint brain regions associated with visual processing. ESRD patients have been reported to exhibit impairments in object recognition and localization (Viggiano et al., 2020) and visual–spatial working memory (Elias et al., 2013; Huang et al., 2021). The superior parietal gyrus and superior occipital gyrus are crucial nodes in the dorsal visual stream (Migliaccio et al., 2016), which is responsible for perceiving object location and spatial motion. Disruptions in this pathway are associated with visuospatial performance impairments observed in ESRD (Bugnicourt et al., 2013). The IFOF connects posterior occipitotemporal regions to frontal lobe regions (Thomas et al., 2008), and its reduced WM integrity is implicated in the disruption of the structural network associated with object recognition between the occipital and temporal lobes (Song et al., 2015). Moreover, visual–spatial working memory has been shown to rely on the collaborative interaction between parietal and occipitotemporal brain regions, particularly with the parietal cortex exhibiting sensitivity to task load and featural complexity (Song et al., 2015). In patients with ESRD, the lower activation levels in the parietal cortex during visual–spatial working memory tasks are associated with CIs (Huang et al., 2021). Therefore, based on the above discussion, our fusion analysis results have revealed the covariant abnormalities related to visuospatial performance impairments in ESRD.

The precentral gyrus in GMV is structurally connected to the CST in FA, where the precentral gyrus is located in the primary motor cortex and is recognized as the essential structure for the execution of voluntary movements (Goodman et al., 2022). However, the precentral gyrus is found to show atrophy or weakened functional connections in patients with MCI in ESRD (Zhang et al., 2013; Mu et al., 2020; Chen et al., 2021). A functional MRI study has also identified the activation of the precentral gyrus in false retrieval of memory and topographic memory (Kurkela and Dennis, 2016). It remains unclear why cognitive performance requires the involvement of the motor cortex (Chen et al., 2020). The CST is a primary WM tract involved in sensory-motor functions (Seo and Jang, 2013) and has been observed to exhibit decreased FA values in ESRD (Hsieh et al., 2009). The covariation of the CST and the precentral gyrus is consistent with the finding of an animal model study that damage to the CST could induce notable alterations in the excitability of the connected motor cortex (Zaaimi et al., 2012). Although the mechanisms linking motor-related brain structures to cognition remain unclear, studies have demonstrated that low motor function is associated with an accelerated decline in cognitive function (Wang et al., 2023b). For example, there is a significant correlation between motor performance in ESRD patients and CIs (Otobe et al., 2019). Therefore, our results may provide potential neuroimaging biomarkers for investigating the relationship between abnormal motor-related brain structures and cognition in ESRD.

In IC2, abnormal white matter integrity was found in the forceps minor, which connects the bilateral medial superior frontal gyrus and is part of the structurally connected network associated with the functional DMN (Luo et al., 2012). Franco et al. considered that the functional connectivity within the DMN highly depends on the integrity of the WM connecting the two hemispheres, particularly the forceps minor (Franco et al., 2008). Impairment of the structurally connected network in the DMN due to abnormalities in the forceps minor may be implicated in cognitive dysfunction (Mamiya et al., 2018). The dorsolateral prefrontal cortex is the highest cortical area responsible for executive functions (Greene et al., 2001), and the orbitofrontal cortex is the primary neural mechanism for human emotional generation (Kringelbach, 2005). Abnormalities in these two brain regions are consistent with Qiu et al.’s VBM results (Qiu et al., 2014). Damage to these regions may lead to dysexecutive syndrome (John, 2009), which is one of the symptoms of MCI in ESRD patients, resulting in reduced function in aspects such as effective control of thinking and behavior, task planning, problem-solving, and strategy selection.

### 4.2 The role of joint neuronal changes in mediating the relationship between CIs and uremic toxins

Results of the mediating effect analysis revealed that the serum phosphate caused the deceased FA value of WM, then the decreased FA value further caused the decreased GMV and finally forced the CI in ESRD. High phosphate levels have been reported to correlate with Alzheimer’s disease (Li et al., 2017). However, the relationship of CI with serum phosphate has not been reported in chronic kidney disease. Our study addresses the possible mechanism (Etgen, 2015). High serum phosphate levels can produce phosphate toxicity, causing increased neuroinflammation, brain cell shrinkage, and apoptosis (Brown, 2020). Increased neuroinflammation can reduce myelin essential protein, change neurofilament expression, reduce structural coherence, and significantly decrease fractional anisotropy on DTI (Jantzie et al., 2020). In addition, high phosphate levels can cause endothelial dysfunction, atherosclerosis, cerebral small vessel disease, etc. (Rroji et al., 2022). The cerebral microvascular dysfunction further causes the decreased FA values of WM (Bagi et al., 2022). WM and GM are two major complementary functional compartments of the brain tissue, where GM is for neuronal cell bodies, and WM belongs to myelinated axonal tracts (Pareek et al., 2018). WM axonal connection and afference are highly correlated with GMV, implying the constructive interrelationship between the WM and GM compartments. Furthermore, the interrelationship was causational and generative, WM augmented axonal afferent functioning enhances the GMV. Therefore, the decreased FA showed a significant positive correlation with decreased GMV, supporting our mediation analysis findings. Finally, the decreased GMV caused the CI in the ESRD (Chai et al., 2015; Wang et al., 2022). This discovery provided new insights into how joint brain changes contribute to cognitive impairment in ESRD, aiding our further understanding of the pathological mechanisms underlying cognitive impairment in ESRD.

## 5 Limitations

The current study has several limitations. First, the multimodal information in our study was limited to local quantitative measures of GMV, FA, and ALFF, the most classic voxel-based mapping of brain structure and function. Additional dimensions such as microstructural quantifications that can specifically characterize neurodegeneration such as fiber demyelination and fiber density mapping, may add extra knowledge to the current study. The fused analysis algorithm could also be further extended to be able to incorporate more abstract types of quantitative measures, such as structural and functional connectivity, graph-based information, etc. Second, the current study design did not include protein-bound uremic toxins such as indoxyl sulfate, p-cresyl sulfate, indole acetic acid, hippuric acid, and kynurenine, among others, which cannot be obtained from conventional clinical blood biochemical tests. Future investigation may benefit from a more comprehensive analysis with a more complicated study design. Third, due to time constraints and the physical condition of patients, this study only collected complete and usable MMSE scores. In subsequent data collection, we aim to use multiple cognitive assessment tools, such as the Montreal Cognitive Assessment (MoCA) and the Clinical Dementia Rating (CDR), to improve the reliability of the study results. Last but not least, the statistical power of both fusion analysis and mediation analysis critically relies upon the sample size. As the project is still ongoing, a larger sample size would greatly strengthen the statistical analyses and conclusions drawn.

## 6 Conclusion

In this study, we used the fusion algorithm mCCA+jICA to reveal the associative altered patterns of GMV, FA, and ALFF of CI in ESRD, providing new insights into the covariant abnormalities in brain structure and function associated with CI. Brain white and gray matter changes mediate the relationship between serum phosphate and CI in ESRD, which provides the possible role of joint brain changes in the relationship between toxins and cognitive impairments.

## Data availability statement

The raw data supporting the conclusions of this article will be made available by the authors, without undue reservation.

## Ethics statement

The studies involving humans were approved by The Ethics Committee of Tianjin First Central Hospital. The studies were conducted in accordance with the local legislation and institutional requirements. The participants provided their written informed consent to participate in this study. Written informed consent was obtained from the individual(s) for the publication of any potentially identifiable images or data included in this article.

## Author contributions

YL: Writing – review & editing, Writing – original draft, Software. HW: Writing – review & editing, Validation. GS: Writing – review & editing, Visualization, Formal analysis. YutC: Writing – review & editing, Investigation, Formal analysis. YonC: Writing – review & editing, Data curation. YuaC: Writing – review & editing, Software, Methodology. JZ: Writing – review & editing, Formal analysis, Data curation. CC: Writing – review & editing, Resources. QF: Writing – review & editing, Supervision, Project administration, Methodology, Funding acquisition, Conceptualization. SX: Writing – review & editing, Resources, Conceptualization.

## Footnotes

^1^http://mialab.mrn.org/software/fit
