# Supplementary material for: The covariant structural and functional neuro-correlates of cognitive impairments in patients with end-stage renal diseases
Source: Front Neurosci. 2024 Apr 15;18:1374948. doi: 10.3389/fnins.2024.1374948 (PMC11056510; doi:10.3389/fnins.2024.1374948)
Supplement: Supplementary file 1 [file Data_Sheet_1.DOCX]

Supplementary Material

# The Preprocessing Pipeline of Multi-Modal Imaging Data

The preprocessing of sMRI data is illustrated in **Fig. S1**. Maps of gray matter (GM) were obtained following white/gray tissue segmentation on the original T1-weighted (T1w) images (Dale et al., 1999). The T1w images were registered to the Montreal Neurological Institute (MNI) template, and the generated deformation field was used to transform GM volume fractions maps to the MNI standard space. The resulting Jacobian determinants were used to modulate the GM volume fraction maps to obtain the GM volume (GMV) maps, followed by the smoothing of GMV maps using a Gaussian kernel of FWHM of 8mm.

| 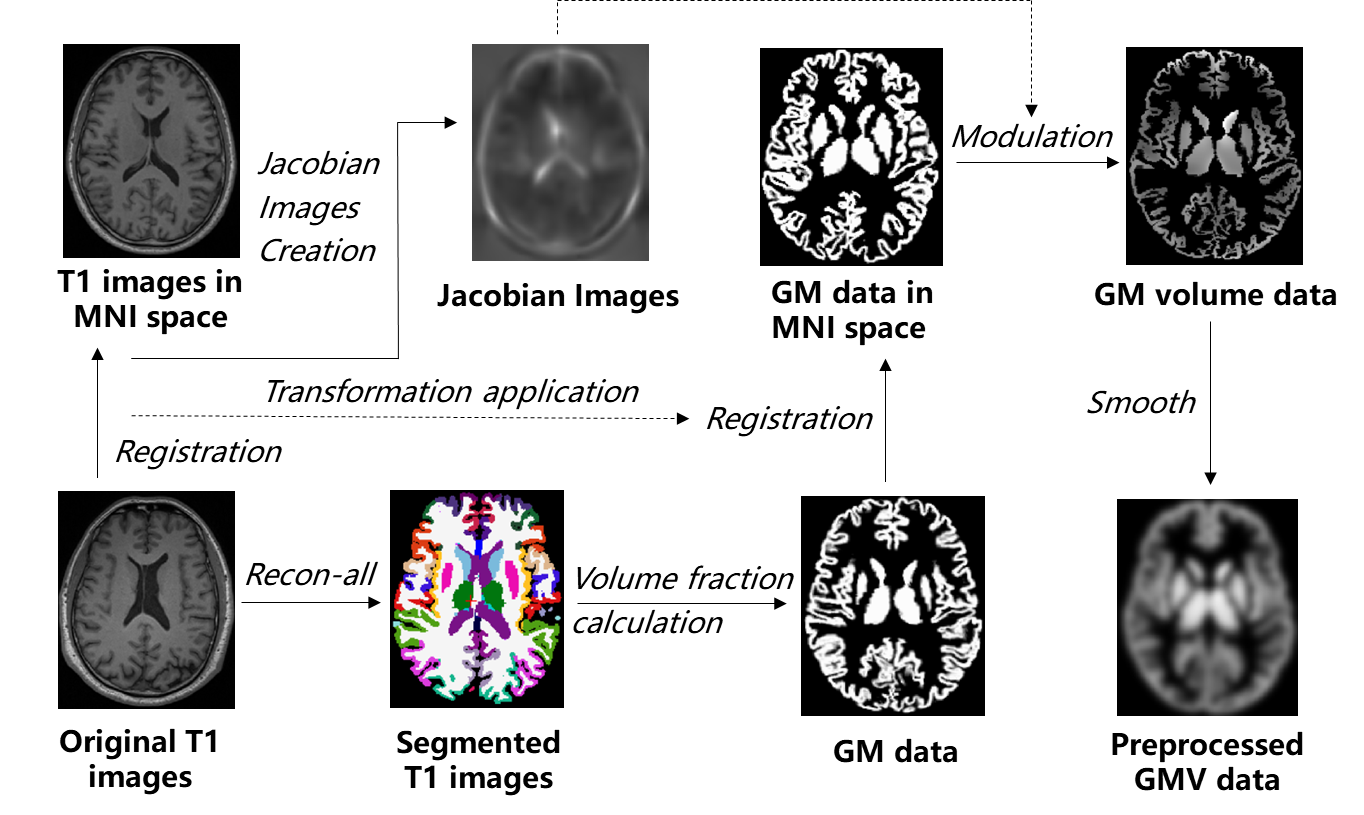 |
| --- |
| **Fig. S1. Workflow of sMRI data processing** |

The preprocessing of dMRI data is illustrated in **Fig. S2**. After eddy current correction, DTI fitting was performed using FMRIB Software Library (FSL) (Jenkinson et al., 2012) to obtain FA maps, which were then registered to the MNI standard space using Advanced Normalization Tools (ANTs) (Avants et al., 2011), followed by smoothing with a Gaussian kernel of FWHM of 8mm.

| 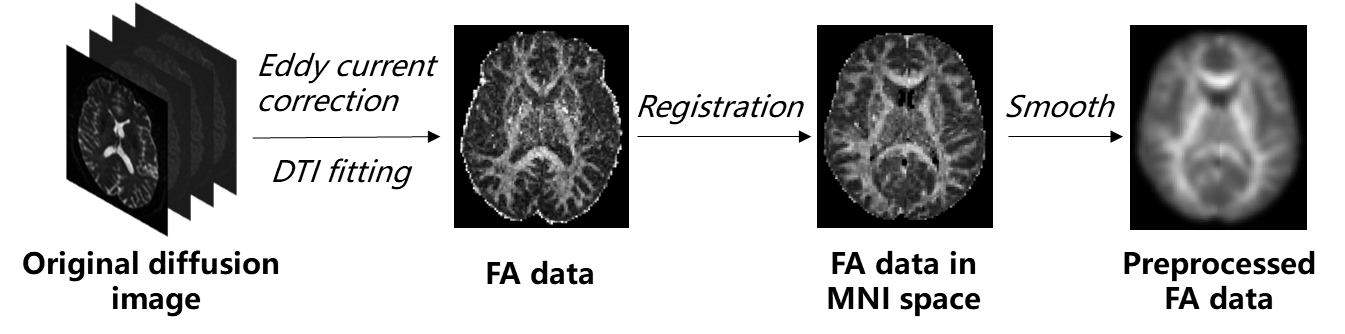 |
| --- |
| **Fig. S2. Workflow of dMRI data processing** |

The preprocessing of fMRI data is illustrated in **Fig. S3**. The original fMRI data was processed by the following steps: 1) removal of the first five time points; 2) slice-timing correction; 3) regression of nuisance signals; 4) bandpass filtering with 0.008-0.08 Hz; 5) global signal regression; and 6) smooth. Finally, the amplitude of low-frequency fluctuations (ALFF) was computed for each voxel, and the ALFF maps were registered to the MNI standard space using ANTs.

| 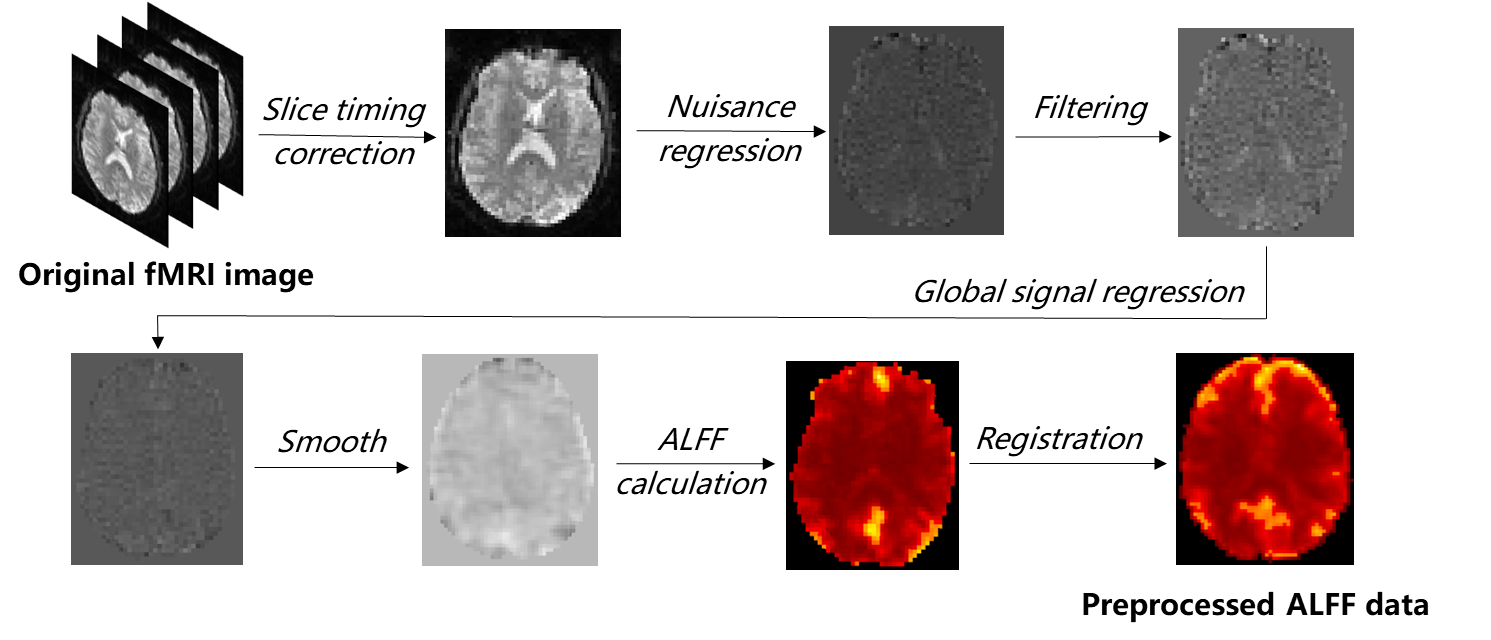 |
| --- |
| **Fig. S3. Workflow of fMRI data processing** |

# Diffusion Fiber Tracking

Diffusion MRI data from one representative healthy subject was used for fiber tracking using DSI_Studio (http://dsi-studio.labsolver.org). DTI streamline fiber tracking was conducted using FA_IC clusters as regions of interest (ROIs), and clusters in the GMV or ALFF modalities within the same IC was used as termination masks. The step size for fiber tracking was set to 2mm, with a threshold of 45° for the turning angle between adjacent voxels, and the tracking was confined to regions where the FA value was greater than 0.3. The fiber density maps were obtained through tractography, representing the total number of tracks passing through each voxel (Calamante et al., 2010), and were subsequently registered to the 2×2×2 mm^3^ MNI space. A threshold of 10 on the fiber density maps was used to report tractography findings, according to the Johns Hopkins white matter tractography atlas (Hua et al., 2008) in FSL.

# Other Group-Discriminative ICs

In addition to the two joint group-discriminative ICs discussed in the main text, 4 ICs were found to reveal inter-group differences in single modalities, including ALFF_IC11, GMV _IC10, and GMV_IC7.

In ALFF_IC11 (**Fig. S5**), the significant brain regions include the cuneus, precuneus, and superior occipital gyrus, which are similar to those observed in ALFF_IC3.

The distribution of abnormal brain regions with altered gray matter volume in GMV_IC10 (**Fig. S6**) includes the middle temporal gyrus, globus pallidus, caudate nucleus, and insula. Notably, the middle temporal gyrus and caudate nucleus are also observed in GM_IC3 and GM_IC2 respectively. The study conducted by Da Silva et al. revealed atrophy in the globus pallidus among ESRD patients undergoing hemodialysis. Bilateral pallidal hyperintensity on T1-weighted images was also observed (da Silva et al., 2007), likely attributed to the accumulation of serum manganese in the central nervous system. Additionally, a voxel-based morphometry study by Zhang et al. demonstrated reduced gray matter volume in the bilateral insulae of patients with ESRD (Zhang et al., 2013).

The significant brain regions identified in GMV_IC7 (**Fig. S7**) include the superior temporal gyrus, middle temporal gyrus, angular gyrus, and precentral gyrus. Among them, the superior temporal gyrus, middle temporal gyrus, and precentral gyrus also exhibit GMV abnormalities in GMV_IC3. Several studies have indicated atrophy or weakened functional connectivity in the angular gyrus of patients with ESRD (Qiu et al., 2014; Jin et al., 2020; Su et al., 2021). The angular gyrus is implicated in the maintenance of memory retrieval and self-awareness, and it plays a crucial role in language functions. The functional abnormalities in the angular gyrus may be associated with language impairments observed in ESRD (Viggiano et al., 2020).

| 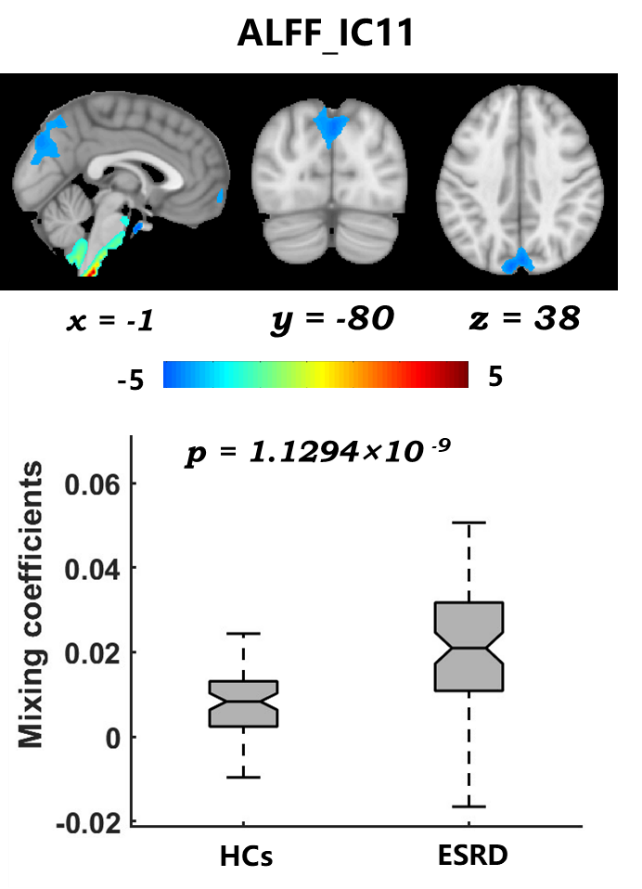 | 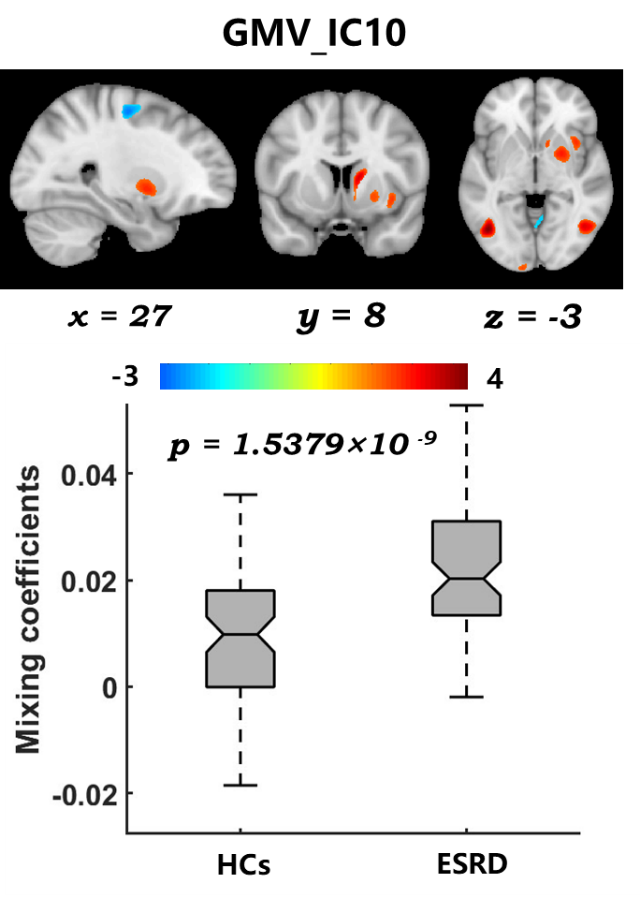 |
| --- | --- |
| **Fig. S5. Map of IC11_ALFF and its corresponding mixing coefficients in two groups**. The IC maps were only presented for \|z\|≥2, and all p-values were corrected for false discovery rate (FDR) | **Fig. S6. Map of IC10_GMV and its corresponding mixing coefficients in two groups**. IC maps were only presented for \|z\|≥2, and all p-values were corrected for false discovery rate (FDR) |

| 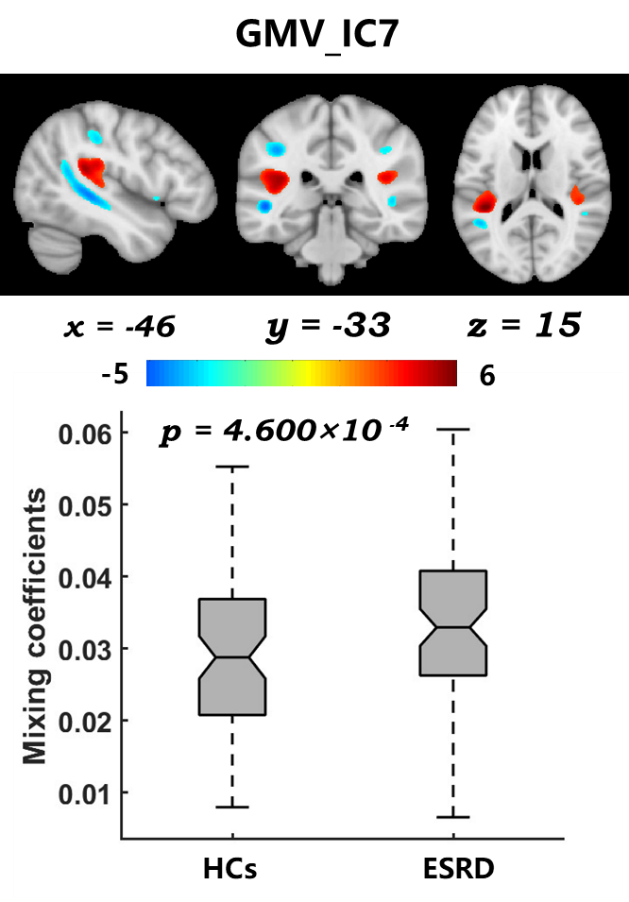 |
| --- |
| **Fig. S7. Map of IC7_GMV and its corresponding mixing coefficients in two groups**. IC maps were only presented for \|z\|≥2, and all p-values were corrected for false discovery rate (FDR) |

# References

Avants, B.B., Tustison, N.J., Song, G., Cook, P.A., Klein, A., and Gee, J.C. (2011). A reproducible evaluation of ANTs similarity metric performance in brain image registration. *Neuroimage* 54(3)**,** 2033-2044. doi: 10.1016/j.neuroimage.2010.09.025.

Calamante, F., Tournier, J.-D., Jackson, G.D., and Connelly, A. (2010). Track-density imaging (TDI): Super-resolution white matter imaging using whole-brain track-density mapping. *NeuroImage* 53(4)**,** 1233-1243. doi: <https://doi.org/10.1016/j.neuroimage.2010.07.024>.

da Silva, C.J., da Rocha, A.J., Jeronymo, S., Mendes, M.F., Milani, F.T., Maia, A.C.M., et al. (2007). A preliminary study revealing a new association in patients undergoing maintenance hemodialysis: manganism symptoms and T1 hyperintense changes in the basal ganglia. *AJNR. American Journal of Neuroradiology* 28(8)**,** 1474-1479.

Dale, A.M., Fischl, B., and Sereno, M.I. (1999). Cortical surface-based analysis. I. Segmentation and surface reconstruction. *Neuroimage* 9(2)**,** 179-194. doi: 10.1006/nimg.1998.0395.

Hua, K., Zhang, J., Wakana, S., Jiang, H., Li, X., Reich, D.S., et al. (2008). Tract probability maps in stereotaxic spaces: analyses of white matter anatomy and tract-specific quantification. *NeuroImage* 39(1)**,** 336-347.

Jenkinson, M., Beckmann, C.F., Behrens, T.E., Woolrich, M.W., and Smith, S.M. (2012). FSL. *Neuroimage* 62(2)**,** 782-790. doi: 10.1016/j.neuroimage.2011.09.015.

Jin, M., Wang, L., Wang, H., Han, X., Diao, Z., Guo, W., et al. (2020). Structural and Functional Alterations in Hemodialysis Patients: A Voxel-Based Morphometry and Functional Connectivity Study. *Front Hum Neurosci* 14**,** 80. doi: 10.3389/fnhum.2020.00080.

Qiu, Y., Lv, X., Su, H., Jiang, G., Li, C., and Tian, J. (2014). Structural and functional brain alterations in end stage renal disease patients on routine hemodialysis: a voxel-based morphometry and resting state functional connectivity study. *PLoS One* 9(5)**,** e98346. doi: 10.1371/journal.pone.0098346.

Su, H., Fu, S., Liu, M., Yin, Y., Hua, K., Meng, S., et al. (2021). Altered Spontaneous Brain Activity and Functional Integration in Hemodialysis Patients With End-Stage Renal Disease. *Front Neurol* 12**,** 801336. doi: 10.3389/fneur.2021.801336.

Viggiano, D., Wagner, C.A., Martino, G., Nedergaard, M., Zoccali, C., Unwin, R., et al. (2020). Mechanisms of cognitive dysfunction in CKD. *Nat Rev Nephrol* 16(8)**,** 452-469. doi: 10.1038/s41581-020-0266-9.

Zhang, L.J., Wen, J., Ni, L., Zhong, J., Liang, X., Zheng, G., et al. (2013). Predominant gray matter volume loss in patients with end-stage renal disease: a voxel-based morphometry study. *Metab Brain Dis* 28(4)**,** 647-654. doi: 10.1007/s11011-013-9438-7.
